# A Comparison of the Clinical and Epidemiological Characteristics of Adult Patients with Laboratory-Confirmed Influenza A or B during the 2011–2012 Influenza Season in Korea: A Multi-Center Study

**DOI:** 10.1371/journal.pone.0062685

**Published:** 2013-05-03

**Authors:** Seong-Heon Wie, Byung Hak So, Joon Young Song, Hee Jin Cheong, Yu Bin Seo, Sung Hyuk Choi, Ji Yun Noh, Ji Hyeon Baek, Jin Soo Lee, Hyo Youl Kim, Young Keun Kim, Won Suk Choi, Jacob Lee, Hye Won Jeong, Woo Joo Kim

**Affiliations:** 1 Division of Infectious Diseases, Department of Internal Medicine, St. Vincent’s Hospital, College of Medicine, The Catholic University of Korea, Seoul, Korea; 2 Department of Emergency Medicine, St. Vincent’s Hospital, College of Medicine, The Catholic University of Korea, Seoul, Korea; 3 Division of Infectious Diseases, Department of Internal Medicine, Korea University Guro Hospital, Korea University College of Medicine, Seoul, Korea; 4 Department of Emergency Medicine, Korea University Guro Hospital, Korea University College of Medicine, Seoul, Korea; 5 Department of Microbiology, Korea University College of Medicine, Seoul, Korea; 6 Division of Infectious Diseases, Department of Internal Medicine, Inha University Hospital, Inha University School of Medicine, Incheon, Korea; 7 Division of Infectious Diseases, Department of Internal Medicine, Wonju Christian Hospital, Wonju College of Medicine, Yonsei University, Wonju, Korea; 8 Division of Infectious Diseases, Department of Internal Medicine, Korea University Ansan Hospital, Korea University College of Medicine, Seoul, Korea; 9 Division of Infectious Diseases, Department of Internal Medicine, Kangnam Sacred Heart Hospital, Hallym University College of Medicine, Seoul, Korea; 10 Division of Infectious Diseases, Department of Internal Medicine, Chungbuk University Hospital, Chungbuk National University College of Medicine, Cheongju, Korea; Tulane School of Public Health and Tropical Medicine, United States of America

## Abstract

**Background:**

During the 2011/2012 winter influenza season in the Republic of Korea, influenza A (H3N2) was the predominant virus in the first peak period of influenza activity during the second half of January 2012. On the other hand, influenza B was the predominant virus in the second peak period of influenza activity during the second half of March 2012. The objectives of this study were to compare the clinical and epidemiological characteristics of patients with laboratory-confirmed influenza A or influenza B.

**Methodology/Principal Findings:**

We analyzed data from 2,129 adult patients with influenza-like illnesses who visited the emergency rooms of seven university hospitals in Korea from October 2011 to May 2012. Of 850 patients with laboratory-confirmed influenza, 656 (77.2%) had influenza A (H3N2), and 194 (22.8%) influenza B. Age, and the frequencies of cardiovascular disorders, diabetes, hypertension were significantly higher in patients with influenza A (H3N2) (*P*<0.05). The frequencies of leukopenia or thrombocytopenia in patients with influenza B at initial presentation were statistically higher than those in patients with influenza A (H3N2) (*P*<0.05). The rate of hospitalization, and length of hospital stay were statistically higher in patients with influenza A (H3N2) (*P*<0.05), and of the 79 hospitalized patients, the frequency of diabetes, hypertension, cases having at least one of the comorbid conditions, and the proportion of elderly were significantly higher in patients with influenza A (H3N2) (*P*<0.05).

**Conclusions:**

The proportion of males to females and elderly population were significantly higher for influenza A (H3N2) patients group compared with influenza B group. Hypertension, diabetes, chronic lung diseases, cardiovascular disorders, and neuromuscular diseases were independently associated with hospitalization due to influenza. Physicians should assess and treat the underlying comorbid conditions as well as influenza viral infections for the appropriate management of patients with influenza.

## Introduction

During the 2011–2012 northern hemisphere winter influenza season, the temperate countries of northern Asia had both influenza A (H3N2) and influenza B peaks, with influenza A (H3N2) appearing first in the Republic of Korea and Japan followed by influenza B, with the reverse sequence occurring in China and Mongolia [1]. In the Republic of Korea, influenza A (H3N2) was the predominant virus in the first peak period of influenza activity during the second half of January 2012. On the other hand, influenza B was the predominant virus in the second peak period of influenza activity during the second half of March 2012 [1], [2].

In the Republic of Korea, Influenza B increased markedly late in the season, even though influenza A (H3N2) was the predominant virus during the 2011–2012 winter influenza season in the northern hemisphere. Moreover, the incidence of influenza B in the 2011–2012 influenza season was much higher than in the 2010–2011 influenza season in the Republic of Korea [2]. It was also reported that newly emerging reassortants between different lineages of influenza B may be associated with unusual influenza B epidemics [3], [4]. Therefore, the increasing proportion of influenza B deserves further analysis and monitoring of both influenza B and influenza A.

As there have been few studies describing the clinical characteristics of influenza B infection or comparing the clinical and epidemiological characteristics of patients with laboratory-confirmed influenza A or influenza B, a prospective, observational cohort comparing the clinical characteristics and clinical outcomes of patients with influenza A or influenza B for the management and control of influenza viral infections in the future is necessary.

We investigated the clinical and demographic data of 850 adult patients with laboratory-confirmed influenza A (H3N2) or influenza B infections who visited the emergency departments of seven university hospitals in the Republic of Korea during the 2011–2012 influenza season and compared the clinical manifestations, laboratory parameters, influenza vaccination rates, hospitalization rates, clinical outcomes, and mortality of the adult patients with influenza A (H3N2) or influenza B infections.

## Materials and Methods

### Study Design

This study was prospectively conducted in seven university hospitals in Korea from 1 October 2011 to 31 May 2012. The hospitals that participated in the study were: Catholic University St. Vincent’s Hospital, Chungbuk National University Hospital, Hallym University Kangnam Sacred Heart Hospital, Inha University Hospital, Korea University Ansan Hospital, Korea University Kuro Hospital, and Wonju Christian Hospital. The Institutional Review Boards (IRBs) of the Catholic University St. Vincent’s Hospital, Chungbuk National University Hospital, Hallym University Kangnam Sacred Heart Hospital, Inha University Hospital, Korea University Ansan Hospital, Korea University Kuro Hospital, and Wonju Christian Hospital have reviewed and approved all protocols of this study (IRB number VC11ONME0118). This study was conducted in accordance with the Declaration of Helsinki and Good Clinical Practices. Written informed consent was obtained from all patients involved in this study.

Surveillance was conducted using the clinical influenza-like illness (ILI) criteria. After obtaining the written informed consent, nasal/throat swabs were taken from patients with ILI. A rapid influenza detection test (SD Bioline Influenza Antigen Test®, Standard Diagnostic, Inc., Korea) was performed bedside and a multiplex respiratory viral polymerase chain reaction test (Seeplex® RV15 ACE Detection, Seegene, Korea) was performed using the nasal/throat swab specimens in the central laboratory.

Influenza patients who were confirmed to have complications or have clinical symptoms limiting physical activities severely were hospitalized. Whether the patients have severe clinical symptoms requiring hospitalization or not was determined by the infectious disease specialist. Thus, the decision to recommend hospitalization for patients with influenza was made by the infectious disease specialist of each participating hospital on the basis of clinical, laboratory, and radiologic findings at initial presentation.

The number of laboratory-confirmed cases and the distribution of influenza types were estimated serially.

### Patient Population

We analyzed data from 2,129 adult patients with ILI. Of these, 656 cases of laboratory-confirmed influenza A (H3N2) and 194 cases of laboratory-confirmed influenza B were enrolled and compared prospectively for clinical symptoms, influenza vaccination history, comorbid conditions, laboratory parameters, and clinical outcomes.

ILI was clinically defined as the sudden onset of fever≥37.8°C and the presence of at least one of the following respiratory symptoms: cough, sore throat, or rhinorrhea/nasal obstruction [5], [6], [7]. Laboratory-confirmed influenza was defined as influenza confirmed by a multiplex respiratory viral polymerase chain reaction (PCR) using the nasal/throat swab specimens. Pneumonia was defined as influenza with pulmonary infiltrate and/or hypoxia. Primary viral pneumonia was defined as pneumonia without documented bacterial pathogen. Combined bacterial pneumonia was defined as pneumonia with isolation of a significant pathogen in respiratory specimen culture or positive result of urinary antigen test. Complications were defined as the spread of influenza virus infection beyond the upper respiratory tract and the involvement of other organs including the lower respiratory tract.

### Data Collection

Surveillance began on October 1, 2011, and the results were reported weekly by an electronic data reporting system throughout the following year.

Data on demographic characteristics, clinical symptoms and relevant physical findings, prior history of influenza vaccination, underlying medical conditions, treatment regimens, laboratory results, days of hospital stay, complications of influenza, and influenza-related mortality were collected for all the eligible patients.

### Reverse Transcriptase-polymerase Chain Reaction (RT-PCR) Assay for Detection of Respiratory Viruses

RNA extraction from the viral transport medium was done using NucliSENS easyMag (BioMérieux, France), and cDNA synthesis was performed using AccuPower® CycleScript RT PreMix (Bioneer, Korea). Multiplex PCR using Seeplex® RV15 ACE Detection (Seegene, Korea) was performed to detect influenza A virus, influenza B virus, adenovirus, coronavirus OC43, coronavirus 229E/NL63, respiratory syncytial virus, rhinovirus, human metapneumovirus, parainfluenza virus, bocavirus, and enterovirus. Samples positive for influenza A virus were tested to differentiate influenza virus subtypes using Seeplex® Influenza A/B Onestep Typing (Seegene, Korea).

### Statistical Methods

SPSS version 21.0 for Windows (SPSS, Inc., Chicago, IL, USA) was used for statistical analysis. Categorical variables were analyzed by a chi-square test or Fisher’s exact test. Continuous variables were analyzed by independent *t*-test or Mann-Whitney test. Logistic regression analysis was performed to evaluate the effects of independent variables on clinical outcomes. A *p*-value of <0.05 by the two-tailed test was considered statistically significant.

## Results

### Demographic and Clinical Characteristics

**Figure 1:**
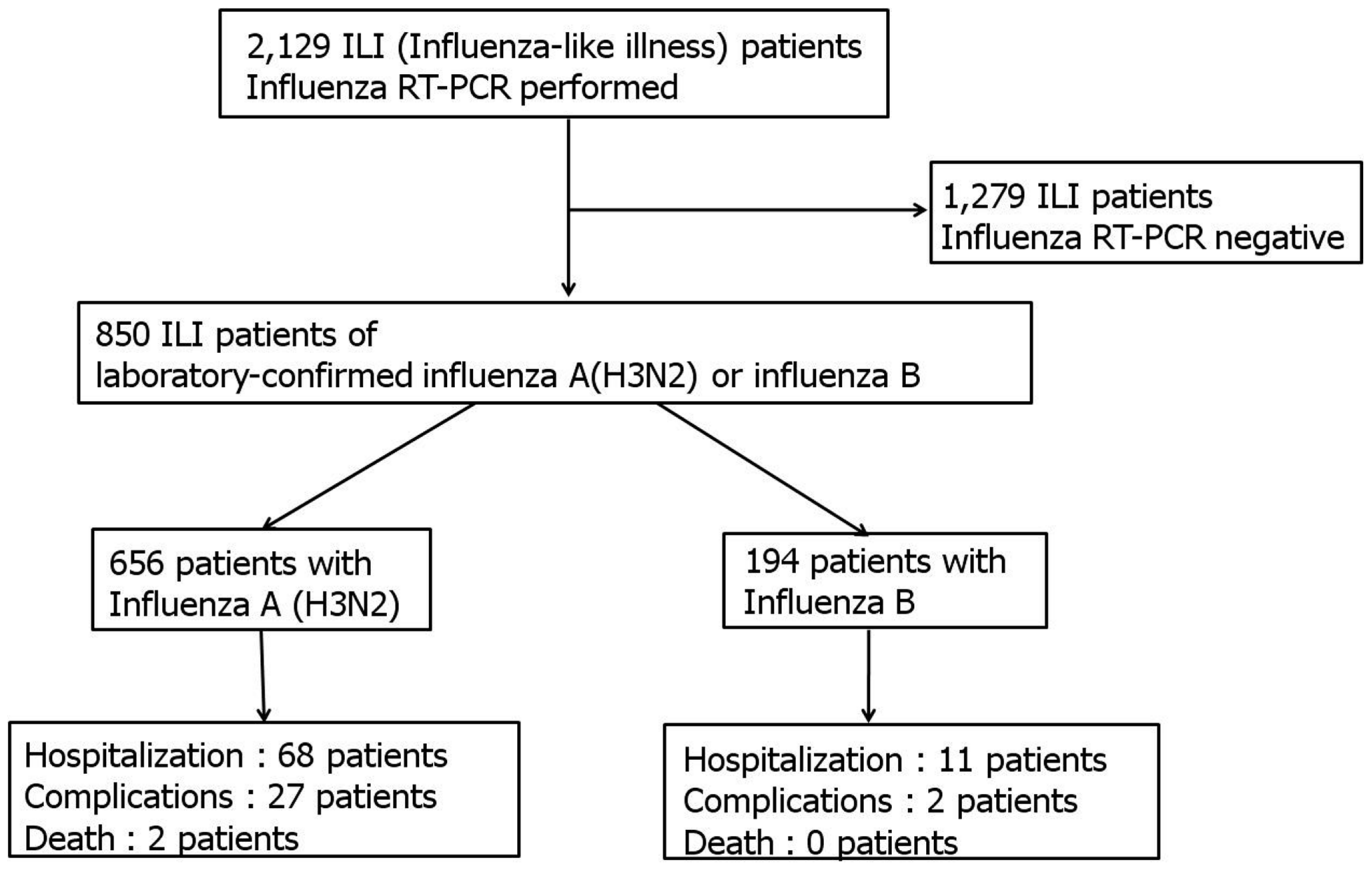
Subjects enrollment and analysis (total influenza-like illnesses).

**Figure 2:**
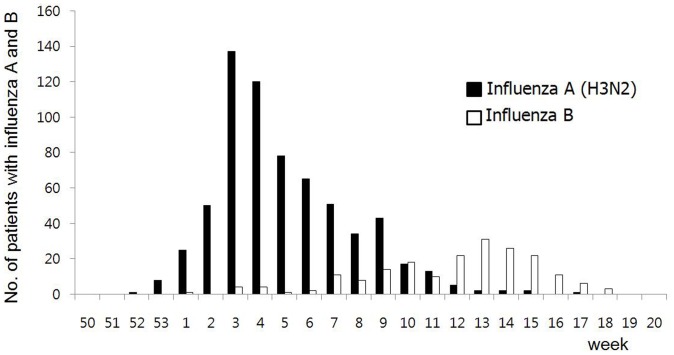
Weekly number of adult patients with laboratory-confirmed influenza A and B, 2011–2012.

A total of 2,129 patients with ILI who visited the emergency rooms of seven university hospitals in Korea from October 2011 to May 2012 were screened. Of these patients, 656 cases of laboratory-confirmed influenza A (H3N2) and 194 cases of laboratory-confirmed influenza B were enrolled and prospectively compared for clinical features, laboratory parameters, complications, and clinical outcomes (Figure 1). During the weeks 41- and 42 (Oct 2∼Oct 15), two female patients were diagnosed with laboratory-confirmed influenza A (H3N2), and then laboratory-confirmed influenza A (H3N2) began to be seen again in week 52 (Dec 18∼Dec 24) and peaked in week 3 (Jan 15∼Jan 21) in the 2011–2012 influenza season. Laboratory-confirmed influenza B began to be seen in week 1 (Jan 1∼Jan 7) and peaked in week 13 (Mar 25∼Mar 31) in the 2011–2012 influenza season, and the number of patients with laboratory-confirmed influenza B was higher than the number of those with laboratory-confirmed influenza A (H3N2) during weeks 12–18 (Mar 18∼May 5) (Figure 2).

Of the 850 patients with influenza virus infection, the number of females was higher than the number of males, and the proportion of female to male patients was significantly higher for influenza B (*P* = 0.016). The mean ages of patients with influenza A (H3N2) and influenza B were 50.1±19.4 and 42.5±16.3 years, respectively, and age was significantly higher in patients with influenza A (H3N2). Underlying medical conditions in these patients included, in descending order of frequency, hypertension, diabetes mellitus, chronic lung diseases, cerebrovascular disorders, cardiovascular disorders, malignancy, pregnancy, chronic hepatic diseases, chronic renal diseases, and neuromuscular diseases. The frequency of cardiovascular disorders, diabetes, and hypertension were significantly higher in patients with influenza A (H3N2). The rates of influenza vaccination in patients with influenza A (H3N2) and B in this season were 25.2% and 17.0%, respectively (Table 1).

**Table 1:** Demographic and epidemiologic characteristics of patients with laboratory-confirmed Influenza A (H3N2) and Influenza B from October 2011 to May 2012.

Characteristics	Influenza A (H3N2)	Influenza B	*P value* *
Number of patients	656	194	
Female gender (%)	380 (57.9)	131 (67.5)	**0.016**
Age (Total), mean years±SD/median	50.1±19.4/47.0	42.5±16.3/39.0	**<0.001**
Age (Female), mean years±SD/median	49.3±19.1/48.0	42.2±16.6/39.0	**<0.001**
Age (Male), mean years±SD/median	51.3±19.9/46.0	43.2±15.7/39.0	**<0.001**
Age range (year)	18–100	18–86	
No. of Age 18–34 (%)	191 (29.1)	71 (36.6)	**0.047**
No. of Age 35–49 (%)	155 (23.6)	57 (29.4)	0.104
No. of Age 50–64 (%)	129 (19.7)	45 (23.2)	0.284
No. of Age 65– (%)	181 (27.6)	21 (10.8)	**<0.001**
Influenza vaccination in 2011–2012 season (%)	165 (25.2)	33 (17.0)	**0.018**
Comorbid conditions, No. (%)			
Cardiovascular disorders	32 (4.9)	1 (0.5)	**0.003**
Cerebrovascular disorders	28 (4.3)	4 (2.1)	0.199
Chronic hepatic diseases	15 (2.3)	3 (1.5)	0.777
Chronic lung diseases	37 (5.6)	4 (2.1)	0.054
Chronic renal diseases	11 (1.7)	2 (1.0)	0.743
Connective tissue disorders	4 (0.6)	4 (2.1)	0.085
Diabetes mellitus	61 (9.3)	9 (4.6)	**0.038**
Hypertension	135 (20.6)	14 (7.2)	**<0.001**
Malignancy	29 (4.4)	3 (1.5)	0.084
Neuromuscular diseases	5 (0.8)	0 (0)	0.594
Organ recipients	1 (0.2)	1 (0.5)	0.405
Pregnancy	17 (2.6)	4 (2.1)	0.798
At least one of the above conditions	243 (37.0)	36 (18.6)	**<0.001**
Not report for the above conditions	413 (63.0)	158 (81.4)	

Data are shown as numbers of patients (% of total) or mean±standard deviation/median as appropriate.

* chi-square test or Fisher’s exact test.

Initial body temperatures in patients with influenza A (H3N2) and influenza B were 38.32±0.76°C and 38.26±0.72°C, respectively (*P* = 0.345). There were no significant differences in the frequency of chills, coughs, crackles, diarrhea, dyspnea, myalgia, seizures, sore throat, sputum, or wheezing (Table 2).

**Table 2:** Clinical symptoms and signs of patients with laboratory-confirmed Influenza A (H3N2) and Influenza B from October 2011 to May 2012.

Clinical symptoms and signs	Influenza A (H3N2), N(%)	Influenza B, N(%)	*P value* *
Number of patients	656	194	
Days from symptom onset to ER visit			
Mean (standard deviation)	1.9 (1.8)	2.1 (2.1)	0.129**
Median (Interquartile range)	1 (1–2)	2(0–3)	
Systemic			
Chill	445 (67.8)	142 (73.2)	0.156
Headache	289 (44.1)	107 (55.2)	**0.006**
General weakness	282 (43.0)	107 (55.2)	**0.003**
Respiratory			
Chest pain	76 (11.6)	38 (19.6)	**0.004**
Cough	593 (90.4)	175 (90.2)	0.937
Crackle	26 (4.0)	4 (2.1)	0.270
Dyspnea	117 (17.8)	35 (18.0)	0.948
Rhinorrhea	402 (61.3)	135 (69.6)	**0.035**
Sore throat	374 (57.0)	123 (63.4)	0.113
Sputum	470 (71.6)	144 (74.2)	0.481
Wheezing	36 (5.5)	7 (3.6)	0.294
Extrarespiratory			
Abdominal pain	40 (6.1)	22 (11.3)	**0.014**
Diarrhea	35 (5.3)	17 (8.8)	0.080
Myalgia	348 (53.0)	116 (59.8)	0.097
Nausea/Vomiting	103 (15.7)	48 (24.7)	**0.004**
Seizure	2 (0.3)	2 (1.0)	0.225

Data are shown as numbers of patients (% of total), unless otherwise indicated.

* chi-square test or Fisher’s exact test, unless otherwise indicated.

** student’s unpaired t-test.

Of the 656 influenza A and 194 influenza B patients, 254 influenza A and 41 influenza B patients had been tested for routine laboratory findings at initial presentation. The mean age of 295 influenza patients who had been tested for routine laboratory findings were higher than that of patients who had not been tested (53.1±19.5 vs. 45.9±18.3 years; *P*<0.001). The frequency of cardiovascular disorders, cerebrovascular disorders, chronic lung diseases, chronic renal diseases, diabetes, and hypertension were statistically higher in patients who had been tested for routine laboratory findings at initial presentation.

Patients with influenza A had statistically higher white blood cell counts, higher platelet counts, and elevated C-reactive protein levels than those with influenza B. Though statistically higher in patients with influenza A, means of white blood cell and platelet counts in patients with influenza A or B were within normal limits. On the other hand, the frequencies of leukopenia and thrombocytopenia in patients with influenza B were statistically higher than those in patients with influenza A (H3N2) (Table 3). There were no significant differences in initial levels of hemoglobin, erythrocyte sedimentation rate, alanine transaminase, aspartate transaminase, bilirubin, albumin, serum creatinine, lactate dehydrogenase, creatine phosphokinase, or prothrombin time (Table 3).

**Table 3:** Routine laboratory findings in patients with laboratory-confirmed Influenza A (H3N2) and Influenza B from October 2011 to May 2012.

Characteristics	Influenza A (H3N2), N = 254, Mean±SD	Influenza B, N = 41, Mean±SD	*P value* *
White blood cell counts (cells/mm^3^)	7,580±3,210	5,606±2,102	**<0.001**
Neutrophile (%)	73.2±11.0	67.8±10.9	**0.007**
Lymphocyte (%)	15.6±9.4	20.0±8.7	**0.006**
Monocyte (%)	10.0±3.6	10.6±3.8	0.311
Frequency of leukopenia (WBC<4,000)	21/254 (8.3%)	11/41 (26.8%)	**<0.001** **
Frequency of leukocytosis (WBC>12,000)	22/254 (8.7%)	0/41 (0%)	0.053**
Hemoglobin (g/dL)	13.4±1.7	13.3±1.2	0.772
Platelet counts (cells/mm^3^)	198,740±63,180	158,275±45,276	**<0.001**
Frequency of thrombocytopenia (PLT<150,000)	54/254 (21.3%)	19/41 (46.3%)	**0.001** **
ESR (mm/hour)	43.2±28.7	21.2±17.0	0.076
CRP (mg/dL)	7.6±14.9	2.9±3.9	**0.049**
AST (IU/L)	25.8±14.4	30.0±14.7	0.102
ALT (IU/L)	22.3±14.9	24.8±19.4	0.364
Bilirubin, total (mg/dL)	0.7±0.6	0.5±0.2	0.065
Albumin (g/dL)	4.1±0.5	4.2±0.3	0.475
Serum creatinine (mg/dL)	1.0±0.6	1.1±0.8	0.415
LDH (IU/L)	235.2±61.7	231.1±47.9	0.696
CPK (IU/L)	138.5±212.1	205.9±391.5	0.130
Prothrombin time (INR)	1.1±0.1	1.1±0.1	0.661
Oxygen saturation (%)	91.0±9.3	96.6±3.6	0.191

Values are means ±SD (standard deviation), unless otherwise indicated.

* student’s unpaired t-test, unless otherwise indicated.

** chi-square test or Fisher’s exact test. AST = aspartate aminotransferase; ALT = alanine aminotransferase; CPK = creatine kinase; CRP = C-reactive protein; ESR = erythrocyte sedimentation rate; LDH = lactate dehydrogenase; INR = international normalized ratio. ESR and oxygen saturation were performed on 39 and 41 of the 254 patients with influenza A (H3N2), and 7 and 6 of the 41 patients with influenza B.

### Clinical Outcomes

The clinical outcomes of the 79 hospitalized patients with laboratory-confirmed influenza A (H3N2) or influenza B are shown in Table 4. The rates of hospitalization of overall 850 patients with influenza A (H3N2) or B were 10.7% (68/656) and 6.2% (11/194), respectively, and the rate of hospitalization, and length of hospital stay were statistically higher in patients with influenza A (H3N2) (*P*<0.05) (Table 4). However, there were no statistically significant differences in the proportion of patients hospitalized for more than 7 or 10 days between influenza A (H3N2) or B patients groups. Of the 79 hospitalized patients, the frequency of diabetes, hypertension, cases having at least one of the comorbid conditions, and the proportion of elderly were significantly higher in patients with influenza A (H3N2) (*P*<0.05). The antiviral agents prescription rates were 100% (68/68) and 90.9% (10/11) for patients hospitalized for influenza A (H3N2) and B, respectively. The antibiotic prescription rates were 52.9% (36/68) and 45.5% (5/11) for patients hospitalized for influenza A (H3N2) and B patients, respectively. Influenza complications such as viral pneumonia, bacterial pneumonia, and acute renal failure occurred in 27 of the 68 hospitalized patients with influenza A (H3N2). However, influenza complications such as viral pneumonia occurred in 2 of the 11 patients hospitalized with influenza B (Table 4).

**Table 4:** Clinical outcomes of hospitalized patients with laboratory-confirmed Influenza A (H3N2) and Influenza B from October 2011 to May 2012.

	Influenza A (H3N2)	Influenza B	*P value* *
No. of hospitalized patients	68	11	
Admission to general ward	65	11	
Admission to intensive care unit	3	0	
Female gender (%)	39 (57.4)	6 (54.5)	0.861
No. of elderly (%)	38 (55.9)	1 (9.1)	**0.007**
Comorbid conditions (%)			
Diabetes mellitus	22 (32.4)	0 (0)	**0.029**
Hypertension	35 (51.5)	0 (0)	**0.001**
At least one of the comorbid conditions	51 (75.0)	3 (27.3)	**0.003**
Hospitalization rate (%)	10.7	6.2	**0.048**
Length of hospital stay, mean day±SD	6.9±4.9	5.3±1.6	**0.038** **
No. of patients hospitalized for more than 7 days (%)	24 (35.3)	3 (27.3)	0.740
No. of patients hospitalized for more than 10 days (%)	14 (20.6)	0 (0)	0.197
Rate of antibiotic use in hospitalized patients (%)	36/68 (52.9)	5/11 (45.5)	0.645
Rate of antiviral use in hospitalized patients (%)	68/68 (100)	10/11 (90.9)	0.139
Antiviral agents used in hospitalized patients			
Oseltamivir, no. of patients	50	10	
Peramivir, no. of patients	16	0	
Oseltamivir+peramivir, no. of patients	2	0	
No. of patients with complication in hospitalized patients (%)	27 (39.7)	2 (18.2)	0.312
No. of patients with viral pneumonia (%)	13 (19.1)	2 (18.2)	
No. of patients with bacterial pneumonia (%)	8 (11.8)		
No. of patients with acute renal failure (%)	2 (2.9)		
No. of patients with other complications (%)	7 (10.3)		
Mortality among total influenza patients (%)	2 (2.9)	0 (0)	1.000

Data are shown as numbers of patients (% of total), unless otherwise indicated.

* chi-square test or Fisher’s exact test, unless otherwise indicated.

** student’s unpaired t-test.

### Factors Associated with Hospitalization due to Laboratory-confirmed Influenza

Among 850 case, the number of hospitalized patients was 79, and that of nonhospitalized patients was 771. In the univariate analysis, the proportion of cases with influenza virus type A (H3N2) (OR 1.92, 95% CI 1.00–3.72; *P* = 0.048), cardiovascular disorders (OR 8.53, 95% CI 4.09–17.79; *P*<0.001), cerebrovascular disorders (OR 4.18, 95% CI 1.86–9.39; *P*<0.001), chronic lung diseases (OR 4.58, 95% CI 2.24–9.39; *P*<0.001), chronic renal diseases (OR 4.52, 95% CI 1.36–15.01; *P* = 0.026), diabetes mellitus (OR 5.81, 95% CI 3.28–10.30; *P*<0.001), hypertension (OR 4.58, 95% CI 2.82–7.46; *P*<0.001), neuromuscular diseases (OR 15.18, 95% CI 2.50–92.25; *P* = 0.007), at least one of comorbid conditions (OR 5.24, 95% CI 3.18–8.63; *P*<0.001) and elderly (OR 3.64, 95% CI 2.27–5.84; *P*<0.001) were statistically significantly higher for hospitalized patients compared with nonhospitalized patients (Table 5).

**Table 5:** Factors associated with hospitalization due to laboratory-confirmed influenza A (H3N2) or B from October 2011 to May 2012.

		Univariate		Multivariate	
Characteristics	No. of hospitalized patients*	OR (95% CI)	*P value*	OR (95% CI)	*P value*
Influenza A (H3N2)	68	1.92 (1.00–3.72)	0.048	1.19 (0.59–2.40)	0.634
Age group (years)					
Non-elderly (18–64)	40	1.0		1.0	
Elderly (65–)	39	3.64 (2.27–5.84)	<0.001	1.07 (0.55–2.07)	0.850
Underlying medical conditions					
Cardiovascular disorders	14	8.53 (4.09–17.79)	<0.001	4.05 (1.72–9.50)	**0.001**
Cerebrovascular disorders	9	4.18 (1.86–9.39)	<0.001	1.42 (0.52–3.85)	0.490
Chronic lung diseases	12	4.58 (2.24–9.39)	<0.001	3.38 (1.51–7.61)	**0.003**
Chronic renal diseases	4	4.52 (1.36–15.01)	0.026	2.15 (0.54–8.56)	0.277
Diabtes mellitus	22	5.81 (3.28–10.30)	<0.001	3.09 (1.57–6.06)	**0.001**
Hypertension	35	4.58 (2.82–7.46)	<0.001	2.37 (1.26–4.47)	**0.008**
Neuromuscular diseases	3	15.18 (2.50–92.25)	0.007	10.18(1.30–79.40)	**0.027**
At least 1 of above conditions	54	5.24 (3.18–8.63)	<0.001		

* Totally, 79 patients were hospitalized. Full model : Influenza A (H3N2), elderly, cardiovascular disorders, cerebrovascular disorders, chronic lung diseases, chronic renal diseases, diabetes mellitus, hypertension, and neuromuscular diseases.

In the multivariate analysis, cardiovascular disorders (OR 4.05, 95% CI 1.72–9.50; *P* = 0.001), chronic lung diseases (OR 3.38, 95% CI 1.51–7.61; *P* = 0.003), hypertension (OR 2.37, 95% CI 1.26–4.47; *P* = 0.008), diabetes mellitus (OR 3.09, 95% CI 1.57–6.06; *P* = 0.001), and neuromuscular diseases (OR 10.18, 95% CI 1.30–79.40; *P* = 0.027) were independently associated with hospitalization of patients with laboratory-confirmed influenza (Table 5).

## Discussion

Influenza is a seasonal acute respiratory infection characterized by fever with upper respiratory symptoms. It is a significant public health problem associated with annual seasonal outbreaks, morbidity, and mortality from influenza-related complications, especially in elderly patients or patients with chronic medical conditions.

Influenza A is classified into subtypes such as H1N1 and H3N2, while influenza B is not classified by subtypes. Influenza A viral infections are associated with more severe disease and with higher pneumonia and influenza mortality [8], [9], [10]. One study in the Netherlands estimated mortality attributable to common viral infections from week 1 of 1999 to week 52 of 2007, and showed that the proportion of deaths attributable to influenza A and B infections with regards to age were 2.0% vs 1.1% (≥85 years), 1.6% vs 0.5 (75–84 years), and 0.8% vs 0% (65–74 years), respectively, with large variations between years [11], [12]. In this study, there is no statistically significant difference in mortality between patients infected with influenza A (H3N2) or B.

The evolutionary rate of the influenza B virus is lower than that of the influenza A virus, and the clinical picture of influenza B is usually milder than influenza A [13], [14], [15]. Influenza A viruses infect humans, birds, pigs, and several other species, and the exchange of genes between different influenza A virus strains may induce the development of new reassortant viruses with pandemic potential. Furthermore, influenza A viruses have different mechanisms to evade early host recognition than influenza B viruses, and can do so more easily. Thus, the early virus-host cell interactions may be different between influenza A and B viruses [16], [17].

It was previously reported in a study analyzing the activation of the antiviral responses of human dendritic cells to influenza A or B viruses that influenza B virus activates the transcription factor interferon (IFN) regulatory factor 3 (IRF3) at a faster rate than influenza A virus. That study showed that the influenza B virus induces an IFN response even if its infectivity is disabled by ultraviolet light. Therefore, initial viral transcription, replication, and viral protein synthesis are unnecessary for influenza B virus-induced antiviral responses, while influenza A viral infections activated IFN responses only after viral RNA synthesis. It is thus proposed that the early influenza virus-host cell interactions differ between influenza A and B viruses [17]. The differences in the early host recognition of the influenza A and B viruses may be associated with different influenza virus pathogenicities, clinical manifestations, and outcomes. The early host recognition of influenza B may contribute to faster and more effective restriction and faster clearance of influenza B viral infections [17].

In this study, patients with influenza A (H3N2) exhibited statistically higher white blood cell counts, platelet counts, C-reactive protein levels, and neutrophile percent in blood than patients with influenza B, however, means of white blood cell and platelet counts in patients with influenza A or B were within normal limits. On the other hand, the frequencies of leukopenia and thrombocytopenia in patients with influenza B were statistically higher than those in patients with influenza A (H3N2), which requires further monitoring and analysis of leukocyte and platelet counts of patients with influenza A or B in the future. Furthermore, we also observed differences in the proportion of females to males and elderly populations between patients infected with influenza A or B, which suggests that the aging and sex ratio of the population might be factors to determine the relative proportion of influenza A and B viral infection in a certain population.

Even though studies have reported that influenza B infections cause severe infection and complications such as pneumonia and death, especially due to bacterial co-infections, the influenza B virus has been overall considered to be less pathogenic and to produce milder symptoms than the influenza A virus in adults [18], [19], [20]. However, influenza virus type (A versus B) was not shown to be a significant independent risk factors of hospitalization in patients with influenza in the multivariate analysis. Moreover, the frequency of underlying comorbid conditions, and the proportion of elderly were lower in patients with influenza B compared to influenza A in this study. Therefore, younger age and the lower frequency of underlying comorbid conditions may be associated with milder clinical courses of patients with influenza B viral infections, while the possibility exists that influenza B virus is less pathogenic. We were also able to confirm that underlying chronic diseases such as cardiovascular disorders, chronic lung diseases, hypertension, diabetes mellitus, and neuromuscular diseases were important factors in determining hospitalization of patients with laboratory-confirmed influenza.

However, this study has some few limitations. First, the influenza cases in this study were a selected sample, as we selected only patients who visited the emergency room. As a result, patients who did not visit emergency rooms were excluded. This could represent a sampling bias. Second, since the sensitivity of RT-PCR was not 100%, true influenza positive patients might be regarded as influenza negative. Third, routine laboratory tests such as white blood cell and blood platelet counts were performed and estimated for only some enrolled patients. Fourth, data regarding comorbid conditions might be underestimated as we only analyzed emergency room medical records. Fifth, we had no data on whether there was mismatch between the different strains of virus isolated and influenza vaccination in the 2011–2012 season, as we did not analyze detailed sequence data.

In conclusion, the proportion of males to females and elderly population were significantly higher for influenza A (H3N2) patients group compared with influenza B group. Hypertension, diabetes, chronic lung disease, cardiovascular disorders, and neuromuscular diseases were independently associated with hospitalization due to laboratory-confirmed influenza A (H3N2) or B, while influenza virus type (A versus B) and elderly were not independent factors associated with hospitalization. Patients with comorbidities (hypertension, diabetes, chronic lung diseases, cardiovascular disorders or neuromuscular diseases) were at greater risk of hospitalization after influenza viral infection. Physicians should assess and treat the underlying comorbid conditions as well as influenza viral infections for the appropriate management of patients with influenza.
